# Recent progress in the synthesis of homotropane alkaloids adaline, euphococcinine and *N*-methyleuphococcinine

**DOI:** 10.3762/bjoc.17.4

**Published:** 2021-01-05

**Authors:** Dimas J P Lima, Antonio E G Santana, Michael A Birkett, Ricardo S Porto

**Affiliations:** 1 Chemistry and Biotechnology Institute, Federal University of Alagoas, 57072970, Maceió, Brazil; 2 Center of Engineering and Agrarian Science, Federal University of Alagoas, 57100-000, Rio Largo, Brazil; 3 Rothamsted Research, West Common, Harpenden, AL5 2JQ, United Kingdon

**Keywords:** 9-azabicyclo[3.3.1]nonane, *Coccinelid* beetles, dipolar cycloaddition, homotropane, ring-closing metathesis

## Abstract

The 9-azabicyclo[3.3.1]nonane ring system is present in several insect- and plant-derived alkaloids. (−)-Adaline (**1**) and (+)-euphococcinine (**2**), found in secretions of *Coccinelid* beetles, and (+)-*N*-methyleuphococcinine (**3**), isolated from the Colorado blue spruce *Picea pungens*, are members of this alkaloid family. Their unique bicyclic system with a quaternary stereocenter, and the potent biological activity exerted by these homotropane alkaloids, make them attractive synthetic targets. This work aims briefly to review the chemical ecology of *Adalia bipunctata* and the recent methodologies to obtain adaline (**1**), euphococcinine (**2**), and *N*-methyleuphococcinine (**3**).

## Introduction

Coccinellid beetles contain a variety of defensive alkaloids that makes them unpleasant for various predators [1]. Over 50 alkaloids have been characterized from ladybirds until now, including perhydroazaphenalenes, aliphatic and aromatic amines, piperidines, pyrrolidines, azamacrolides, dimeric alkaloids and homotropanes [2]. The majority of these alkaloids have an endogenous origin. In a dangerous situation or predator attack, the beetles can emit droplets of hemolymph. This substance comes from the tibiofemoral joints situated in their legs, a mechanism known as reflex bleeding. This situation brings the alkaloids to the surface as an early warning signal to the attacker.

**Figure 1:**
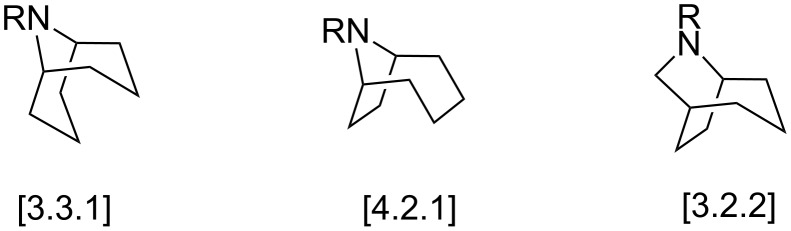
Homotropane (azabicyclononane) systems.

The fluid toxicity and bitterness, added to the characteristic odor of these insects, have been regarded as a protection against insect or vertebrate predators [3]. Bicyclic ring systems bearing a nitrogen bridge are often found in nature [4–6]. Typical examples include cocaine, atropine, and scopolamine. These compounds are 8-azabicyclo[3.2.1]octane derivatives and belong to a large class of natural products known as tropane alkaloids [7–9]. In contrast to tropane alkaloids, the higher homologs homotropanes (9-azabicyclononanes) are less common in nature, but not less important. They possess biological properties, such as nicotinic acetylcholine receptor (nAChR) ligand [10–11], CNS (central nervous system) activity [12–13], and chemical defense [14–16]. Structurally, homotropane alkaloids have different skeletons including [3.3.1], [4.2.1] and [3.2.2] (Figure 1).

**Figure 2:**
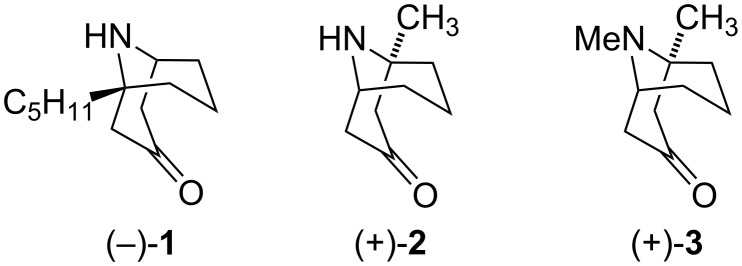
Alkaloids (−)-adaline (**1**), (+)-euphococcinine (**2**) and (+)-*N*-methyleuphococcinine (**3**).

The homotropane alkaloid (−)-adaline (**1**) was isolated from ladybird *Adalia bipunctata* [17] and *Cryptolaemus moutrouzieri* secretions [18]. A methyl analog, (+)-euphococcinine (**2**), has been found in vegetable and animal kingdoms. The compound was first isolated from the Australian coastal plant *Euphorbia atoto* [19], and it is also present in the defense secretion of ladybirds *Cryptolaemus montrouzieri* [18] and *Epilachna varivestis* [16]. Also, (+)-*N*-methyleuphococcinine (**3**) has been identified as a trace homotropane alkaloid isolated from the spruce tree *Picea pungens* [20] (Figure 2)*.*

The small amount of these homotropane alkaloids isolated from ladybirds (e.g., for (−)-adaline, 35 mg from 800 specimens) emphasizes the desirability of practical syntheses for further biological studies [17]. Besides, the attractive structural features of (−)-adaline (**1**) and its relatives have provided new opportunities to develop synthetic strategies [21–23]. Structurally, it has an unsymmetrical bicyclic arrangement, incorporating a secondary amine and bearing a quaternary center. Since the pioneering Tursch’s work [24], a variety of approaches to obtain these alkaloids have been described by many research groups [25–28]. King and Meinwald earlier reviewed some of these syntheses in an elegant approach to coccinellids chemistry and biology [29].

The current work reports a brief description of the chemical ecology of *Adalia bipunctata*. Then we present an up to date review of the synthetic strategies to obtain alkaloids **1**–**3**, including racemic and asymmetric syntheses, aiming to achieve a deep and comprehensive understanding of the area. It also provides suggestions for future studies on homotropane alkaloids. The present review is chronologically organized, encompassing all synthetic works published in the last 25 years.

## Review

### Chemical ecology of *Adalia bipunctata*

Individuals of *Adalia bipunctata* species (2-spot ladybird) display aposematic coloration reinforced by the production and release of remarkable amounts of reflex-fluid, in response to predator attack [29–32]. This liquid can be over 20% of the body weight, in some cases. The amount of the toxic alkaloid (−)-adaline varies between 5–6% of the wet weight of reflex fluid in 2-spot ladybirds. However, the concentration of (−)-adaline found in *A. bipunctata* is about 6–8 times greater than the concentration of coccineline found in *C. septempunctata* [31]. This difference may occur to compensate (−)-adaline's lower toxicity than coccineline. Biosynthetic studies carried out by Laurent et al. [33–34] showed that adults of *A. bipunctata* incubated in vitro with [14,14,14-^3^H_3_]myristic acid incorporated this precursor in (−)-adaline, supporting fatty acid origin for this alkaloid.

Reflex bleeding is costly due to energy expended in chemical synthesis and fluid loss. Therefore, it is only deployed when other strategies have failed, and the ladybird is in severe danger [35–36]. A massive discrepancy in (−)-adaline concentration and reflex-fluid amount can be found within beetles. It suggests that internal aspects such as genetic factors may determine how much energy is invested in chemical defense [30]. Paul et al. [37] demonstrated that parental effects could play a crucial role in determining the color and toxin [(−)-adaline] content of *A. bipunctata* eggs, once the maternal and paternal aposematic phenotype had the most significant effect on egg traits if compared to the maternal responses to offspring predators. Thus, the phenotype can also contribute to the aposematic signal variation in a ladybird’s early life, in addition to genetic factors. In this way, it should consequently lead to success in the species’ survival.

Recent elegant studies by Steele et al. [38–39] provide an insight into the impact of pathogen infection upon production of the alkaloid **1** in *A. bipunctata.* When *A. bipunctata* was infected by the microsporidian pathogen *Nosema adaliae*, larval development was significantly delayed. At elevated temperatures, developmental delays caused by infection were reduced, spore counts and infection decreased, and there was an increase in the content of **1** [38]. In a second study, the authors evaluated the effects of the *N. adaliae* infection and food availability on production of **1** [39]. Infected *A. bipunctata* were shown to produce more **1** than uninfected adults. Furthermore, the daily fed adults produced more of **1** than those adults that were fed irregularly, and uninfected adults that fed irregularly had the lowest content of **1**. The infection load of adults was significantly increased in beetles that were fed irregularly. Taken together, these results suggest that **1** may provide *A. bipunctata* with chemical defence against pathogen challenge.

### Syntheses

#### A concise overview of the strategies towards the synthesis of homotropane alkaloids: before 1995

**Scheme 1:**
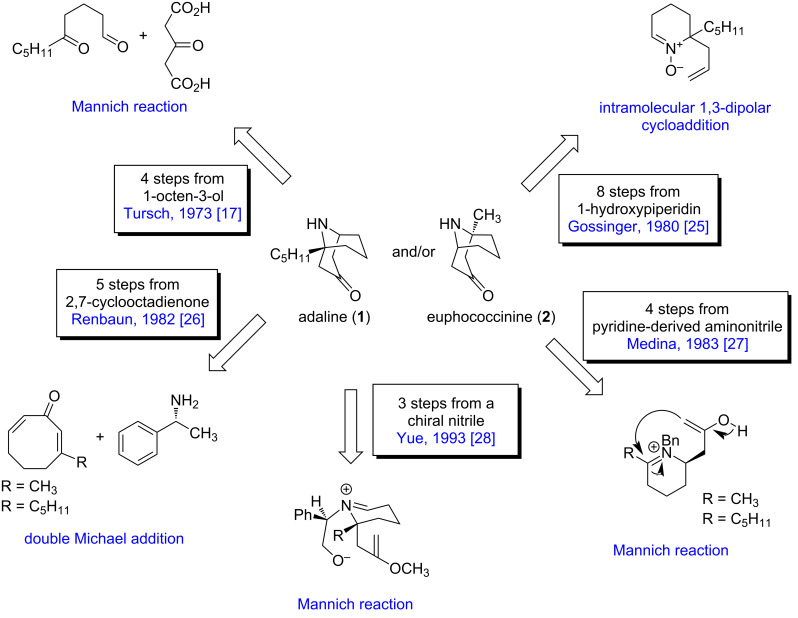
Synthetic strategies before 1995.

As previously reported by King and Meinwald [29], synthetic strategies have been designed and employed in the synthesis of adaline (**1**) and euphococcinine (**2**), before 1995. In brief, **1** and **2** were synthesized in both racemic and asymmetric forms. Key (homotropane construction) steps included: i) inter and intramolecular Mannich reaction; ii) double Michael addition in the cyclooctadienone derivative; and iii) intramolecular 1,3-dipolar cycloaddition. As shown in Scheme 1, the azabicyclononane ring is an interesting target for strategies based on the Mannich reaction. This methodology is mostly used in some cases, with few steps, and from commercially available reagents.

#### Holmes synthesis – 1995

Davison and Holmes prepared racemic (±)-adaline (**1**) and (±)-euphococcinine (**2**). The key step in the synthesis involved the intramolecular dipolar cycloaddition to produce tricyclic isoxazolidines [40].

**Scheme 2:**
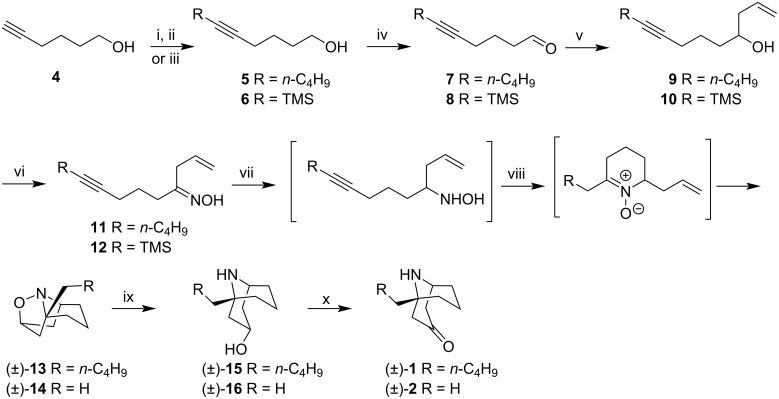
Synthesis (±)-adaline (**1**) and (±)-euphococcinine (**2**). Reagents and conditions: i) 1. dihydropyran, amberlyst-15 resin; 2. *n*-BuLi, TMEDA, then BuBr, 60% over 2 steps; ii) amberlyst-15 resin, MeOH; iii) 1. *n*-BuLi; 2. TMSCl, 3. HCl aqueous, 76% over 3 steps; iv) oxalyl chloride, DMSO, Et_3_N, 80–90%; v) CH_2_=CHCH_2_MgBr, Et_2_O, 70–81%; vi) 1. CrO_3_, HOAc; 2. NH_2_OH·HCl, Py–EtOH, 58–66%; vii) NaCNBH_3_, MeOH, pH 3–4; viii) toluene, reflux, 9 h, 71–76%; ix) Raney-Ni, H_2_, 90 min, 93–96%; x) PCC, CH_2_Cl_2_, 70–72%.

The synthesis started from 5-hexyn-1-ol (**4**, Scheme 2). The alcohol was treated with dihydropyran followed by alkylation using butyllithium and then, acetal deprotection, providing the alcohol **5** as a key starting compound for the (±)-adaline (**1**). Alternatively, the (±)-euphococcinine precursor **6** was prepared from **4**, via deprotonation, silylation, and finally, silyl ether cleavage. Swern oxidation of alcohols **5** and **6** gave aldehydes **7** and **8**, treated with allylmagnesium bromide, to generate secondary alcohols **9** and **10**. These alcohols were converted to oximes **11** and **12** via oxidation with chromium trioxide followed by treatment with hydroxylamine hydrochloride. **11** and **12** were reduced by sodium cyanoborohydride and the resulting hydroxylamines were converted in nitrones, after heating under reflux. These nitrones were not isolated but subjected to intramolecular dipolar cycloaddition to give racemic adducts (±)-**13** and (±)-**14**, with good yields. The synthesis was complete according to the procedure used by Gössinger [25]. Thus, the reductive cleavage of the N–O bond in the presence of Raney-Ni and hydrogen provided the bicyclic alcohols (±)-**15** and (±)-**16**, which were oxidized with pyridinium chlorochromate giving the alkaloids (±)-adaline (**1**) and (±)-euphococcinine (**2**), respectively.

The synthetic route performed by the authors allowed accessing both racemic homotropane alkaloids in 8 steps, starting from alcohol **5** (or **6**) in 15.0–25.3% overall yields. A relevant consideration in Holmes’s synthesis is: the nitrone is the same common intermediate as Gössinger’s [25], which is cyclized to form the tricyclic adducts. While the Gössinger route started from the cyclic 1-hydroxypiperidine, Holmes performed in situ cyclization to prepare the nitrone.

#### Murahashi synthesis – 2000

**Scheme 3:**
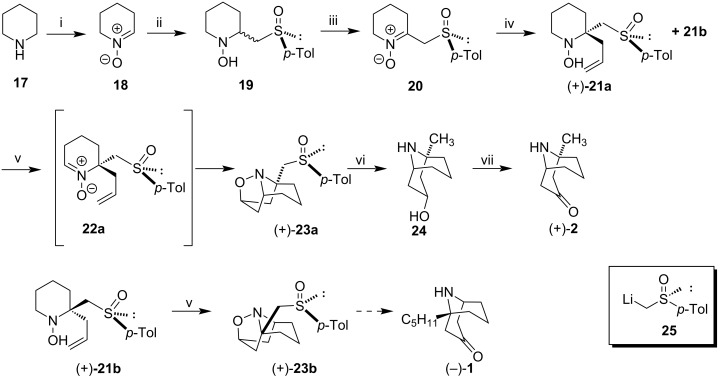
Synthesis (+)-euphococcinine (**2**). Reagents and conditions: i) H_2_O_2_, SeO_2_ (cat), acetone, rt, 88%; ii) **25**, THF, −78 °C, 52% (67:33 diastereomeric mixture); iii) H_2_O_2_, FIEt^+.^ClO_4_ (cat.), MeOH, 0 °C, 55%; iv) AlCl_3_, CH_2_=CHCH_2_MgBr, THF, −78 °C, 54%; v) Ni_2_O_3_, CHCl_3_, rt, 54%; vi) Raney-Ni (W-2), H_2_O, 30 °C, 95%; vii) PCC, CH_2_Cl_2_, rt, 30%.

Starting from secondary amines, Murahashi et al. developed a method for preparing homochiral β-sulfinyl nitrones [41]. Accordingly, (+)-euphococcinine (**2**) was prepared through allylation followed by the 1,3-dipolar cycloaddition of β-sulfinyl nitrone **20** derived from piperidine (**17**). The synthetic sequence performed by the authors is described in Scheme 3. Oxidation of **17** in the presence of hydrogen peroxide, catalyzed by selenium dioxide provided tetrahydropyridine *N*-oxide **18** in 88% yield. **18** was treated with (*R*)-*p*-tolylsulfinylmethyllithium **25** in THF at −78 °C to provide β-sulfinyl hydroxylamine **19** in a diastereoisomeric ratio of 67:33 in 52% yield. Oxidation of **19** to nitrone **20** occurred chemoselectivelly through treatment with a solution of hydrogen peroxide in 3 mol % of 5-ethyluminiflavin perchlorate (FIEt^+.^ClO_4_) as a catalyst in 55% yield. The reaction of β-sulfinyl nitrone **20** with allylmagnesium bromide in the presence of AlCl_3_ provided a mixture of allylpiperidines (+)-**21a** and its isomer (+)-**21b** with 54% and 6% yield, respectively. The treatment of (+)-**21a** with nickel(III) oxide followed by dipolar cycloaddition of the resulting nitrone **22a,** furnished the azatricyclo[3.3.1.1]decane (+)-**23a** in 54% yield. Treatment of (+)-**23a** with Raney nickel resulted in the cleavage of the sulfinyl group and the N–O bond, providing the bicyclic alcohol **24**, which was oxidized with PCC to give (+)-euphococcinine (**2**). The same protocol was applied to (+)-**21b,** furnishing the tricyclic adduct (+)-**23b**, a precursor to (−)-adaline (**1**).

This methodology, based on the synthesis of optically active β-sulfinyl nitrones, was proved to be efficient in the synthesis of (+)-euphococcinine (**2**) in 7 steps from piperidine (**17**), in an overall yield of 2.1%. Specific rotation for (+)-**2** was [α]_D_^24^ +7.43 (*c* 0.350, MeOH); {lit. [α]_D_ +7.5 (*c* 2.0, MeOH), [26]}. The (−)-adaline precursor (+)-**23b** was also accessed from **17** in 5 steps, in an overall yield of 7.3%. Inspired by Gössinger’s work, Murahashi et al. prepared a nitrone from a cyclic amine. However, the route was improved by the introduction of a chiral auxiliary. Despite having a common intermediate, Murahashi's strategy differed from Gössinger's and Holmes' syntheses by being an asymmetric version.

#### Meyers synthesis – 2000

**Scheme 4:**
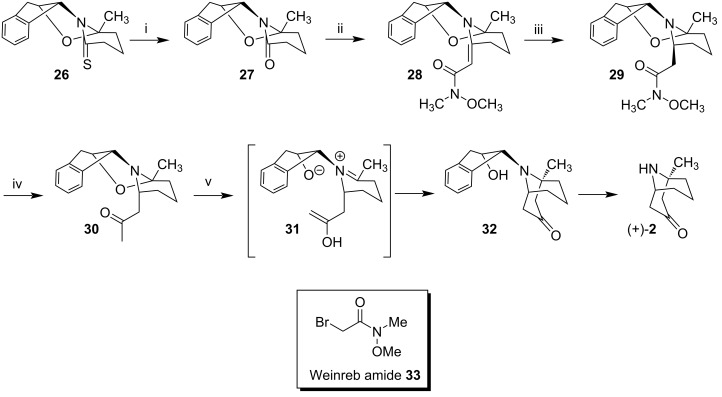
Synthesis (+)-euphococcinine (**2**). Reagents and conditions: i) 2,4-bis(4-phenoxyphenyl)-1,3-dithia-2,4-diphosphetane 2,4-disulfide (Belleau’s reagent), 100%; ii) **33**, triethylamine, trimethyl phosphite, reflux, 69%; iii) H_2_ (60 psi), Pt/C, Na_2_CO_3_, Et_2_OAc, EtOAc, 96%; iv) methyllithium, −78 °C, 85%; v) HOAc/EtOH 1:1, ammonium acetate, 75 °C, overnight, 91%.

Mechelke and Meyers prepared (+)-euphococcinine (**2**) from the bicyclic thiolactam **26** [42]. The strategy was based on an intramolecular Mannich reaction that occurred in intermediate **31** (Scheme 4). Thus, thiolactam **26** was quantitatively converted to lactam **27**, using the Belleau's reagent [43]. **27** was treated with Weinreb amide **33**, prepared from commercially available bromoacetyl bromide [44] to provide the intermediate thioiminium salt, which was heated under the reflux of triethylamine and trimethyl phosphite, providing compound **28** in 69% yield.

The catalytic hydrogenation of **28** occurred in platinum (H_2_, Pt/C) under a pressure of 60 psi of hydrogen (about 4 atm), resulting in amide **29** in 96% yield. This hydrogenation occurred with high stereoselectivity producing a single diastereoisomer of **29**. Then, the amide was treated with methyllithium at −78 °C to provide ketone **30** in 85% yield. Subsequently, the intramolecular Mannich reaction was carried out, leading to the desired alkaloid, via precursor **32**. Ketone **30** was then dissolved in acetic acid/ethanol 1:1 and treated with ten equivalents of ammonium acetate, stirred overnight at a temperature of 75 °C. Work-up followed by chromatographic column purification of the reaction mixture furnished (+)-euphococcinine (**2**) in 91% yield. This single step procedure from **30** not only led to the formation of the bicyclic system but also resulted in the loss of the chiral auxiliary, providing (+)-euphococcinine (**2**).

Meyer's approach led to (+)-euphococcinine (**2**) in 5 steps from lactam **26** in an overall yield of 51.2%. The spectral analysis (^1^H and ^13^C NMR, IR, MS) was identical to that of the natural product [28]. The specific rotation [α]_D_ of +5.7 was also compatible with that found in the literature {lit. [α]_D_ +6 (c 2.0, MeOH), [19]}. Finally, the synthetic sample obtained by the authors when treated with (*S*)–Mosher's acid chloride was converted entirely to a Mosher amide, confirming to be a sample with a high level of enantiomeric purity. As in Murahashi’s synthesis, Meyers also utilized a chiral auxiliary for asymmetric induction. Nonetheless, this method differed from Murahashi's by presenting a diastereoselective intramolecular Mannich cyclization to form the desired homotropane.

#### Ikeda synthesis – 2002

Ikeda et al. prepared azabicycle (±)-**42**, a protected form of (±)-euphococcinine (**2**) [45]. The author’s method focused on the radical reaction of 2-(but-3-ynyl)piperidine (±)-**34** mediated by Bu_3_SnH. This "6-*exo-dig*" cyclization occurred in a regioselective way to provide the 9-azabicyclo[3.3.1]nonane system observed in (±)-**35**.

**Scheme 5:**
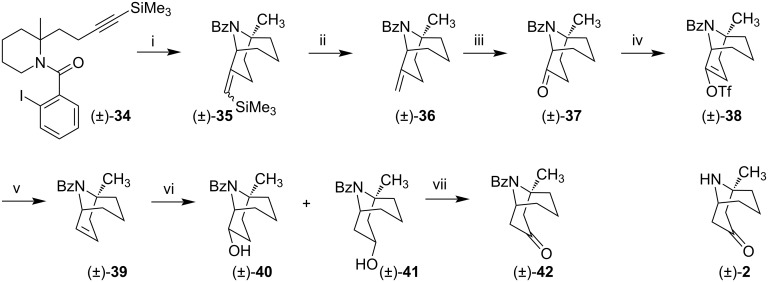
Synthesis of (±)-euphococcinine precursor (±)-**42**. Reagents and conditions: i) Bu_3_SnH, AIBN, toluene, reflux, 95%; ii) CF**_3_**CO**_2_**H, CH**_2_**Cl**_2,_** 89%; iii) O**_3_**, CH**_2_**Cl**_2_**, 78 °C, then PPh**_3,_** 71%; iv) LDA, THF, −78 °C; then Comins’ reagent, 63%; v) Me_2_NHBH_3_, cat. Pd(PPh_3_)_4_, K_2_CO_3_, MeCN, 40 °C, 97%; vi) BH_3_^.^THF, then aq NaOH, H_2_O_2_, 31% (for (±)-**40**) and 39% (for (±)-**41**); vii) TPAP, NMO, 4 Å MS, CH_2_Cl_2,_ 100%.

The precursor (±)-**34** was prepared from *N*-boc-pipecolinate, following a methodology previously described by the authors [46]. The radical Bu_3_SnH-mediated cyclization of (±)-**34,** occurred efficiently to provide (±)-**35** with 95% yield in a 1:1 diastereoisomeric mixture, converted to ketone (±)-**37** via methylene derivative (±)-**36,** in 63% over two steps (Scheme 5). Ketone (±)-**37** was converted to alkenyl triflate (±)-**38** after treatment with LDA at −78 °C, followed by the Comins reagent [47]. (±)-**38** was subjected to palladium-catalyzed hydrogenation conditions to afford alkene (±)-**39**. Hydroboration of (±)-**39** with the borane–THF complex followed by oxidation of the obtained intermediate led to the mixture of alcohols (±)-**40** + (±)-**41** with yields of 31% and 39%, respectively. The ^1^H NMR spectrum confirmed the structure and stereochemistry of alcohol (±)-**41**, where the axial proton in position 3 appears in δ 4.62 as a triplet of triplets, having coupling constants 11.0 and 6.4 Hz. Oxidation of (±)-**41** with the TPAP-NMO system produced ketone (±)-**42,** a potential precursor to (±)-euphococcinine (**2**).

Although being racemic, Ikeda's synthesis employed an innovative “6-*exo-dig”* cyclization to achieve the azabicyclic system. The route accomplished by Ikeda et al. led to the (±)-euphococcinine Bz-protected (±)-**42** in 7 steps from the precursor 2-(but-3-ynyl)piperidine (±)-**34**, in an overall yield of 14.3%. The radical translocation and 6-*exo-dig* cyclization developed by the authors conferred an excellent methodology to obtain the 9-azabicyclo[3.3.1]nonane ring present in (±)-euphococcinine (**2**).

#### Kibayashi synthesis – 2002

**Scheme 6:**
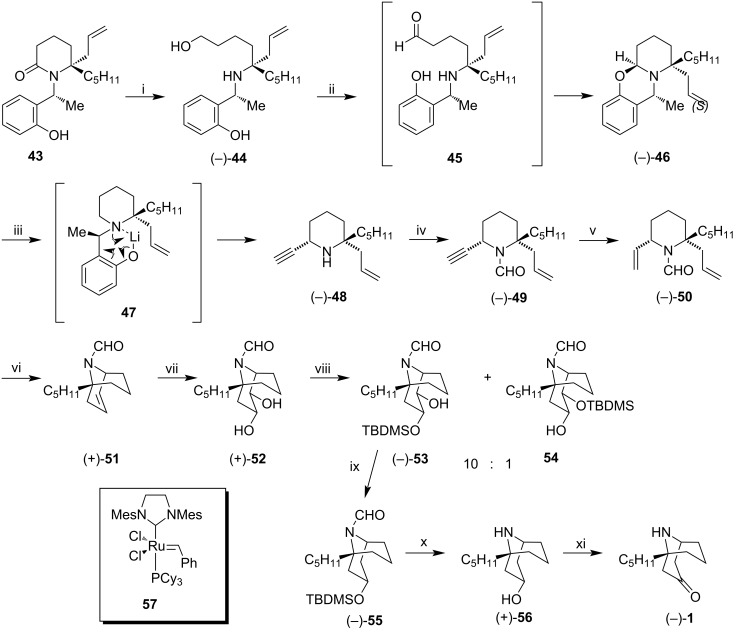
Synthesis of (−)-adaline (**1**). Reagents and conditions: i) LiH_2_NBH_3_, THF, 40 °C, 88%; ii) TPAP, NMO, MeCN, 4A MS, rt, 80%; iii) HC≡CLi^.^H_2_NCH_2_CH_2_NH_2_ (5 equiv), THF, 40 °C, 88%; iv) HCl/MeOH, then HC(OMe)_3_, 93%; v) Lindlar catalyst, H_2_, MeOH, 92%; vi) Grubbs catalyst **57** (0.15 equiv), benzene, 50 °C, 99%; vii) OsO_4_, NMO, MeCN/H_2_O, 97%; viii) TBDMSOTf, Et_3_N, CH_2_Cl_2_, 98%; ix) 1. CS_2_, NaH, MeI, THF; 2. AIBN, Bu_3_SnH, benzene, reflux, 82% over two steps; x) 1. TBAF, THF; 2. Li^.^H_2_NCH_2_CH_2_NH_2_, 90% over two steps; xi) PCC, CH_2_Cl_2_, 77%.

Kibayashi et al. performed the enantioselective synthesis of (−)-adaline (**1**). Their approach had as key steps S_N_2-type alkynylation, activated by lithium ion in a tricyclic N,O-acetal (−)-**46,** and an olefin metathesis (RCM) of a dialkenylpiperidine (−)-**50** for the construction of an azabicyclononane system [48]. The synthetic sequence described by the authors is shown in Scheme 6. The lactam present in **43** was opened by treatment with LiH_2_NBH_3_ in THF at 40 °C to provide amino alcohol (−)-**44** in 88% yield. This amino alcohol underwent cyclization through a one-pot process in the presence of TPAP-NMO, which involved oxidation in generated aldehyde **45**, followed by dehydrocondensation leading to *N*,*O*-tricyclic acetal (−)-**46** in 80% yield. After the treatment of (−)-**46** with the lithium acetylide ethylenediamine complex in THF, a nucleophilic alkynylation occurred, with a reversal of configuration in the reaction center. Then, removal of the 1-(2-hydroxyphenyl)ethyl group via cleavage of the C–N bond, leading to (6*S*)-ethynylpiperidine (−)-**48,** in 88% yield, as a single diastereoisomer. Then, (−)-**48** was treated with trimethyl orthoformate to provide formamide (−)-**49**, being converted to *cis*-2,6-dialkenylformamide (−)-**50** by treatment with the Lindlar catalyst. (−)-**50** underwent a ring-closing metathesis efficiently in the presence of the second generation Grubbs catalyst **57** in 99% yield. The azabicyclic system (+)-**51** underwent dihydroxylation with the OsO_4_-NMO system to form diol (+)-**52** as the only product in 97% yield. Diol (+)-**52** was regioselectively protected in the presence of *tert*-butyldimethylsilane triflate, and triethylamine providing a mixture of monoprotected diols (−)-**53** and **54** in a 10:1 ratio for the least sterically hindered alcohol in 98% yield. After chromatographic separation, (−)-**53** underwent radical-induced Barton–McCombie deoxygenation (AIBN, Bu_3_SnH) to form (−)-**55** in 82% yield. The double deprotection of (−)-**55** occurred by removing the TBDMS and formyl (CHO) groups via treatment with TBAF in dry THF and lithium–ethylenediamine complex, respectively. Finally, the resulting alcohol (+)-**56** was oxidized in the presence of PCC to provide (−)-adaline (**1**).

In this work, the quaternary center was successfully generated before the key cyclization step. It was also the first example of olefin metathesis in (−)-adaline (**1**) synthesis. Kibayashi's approach consisted of 13 steps in an overall yield of about 28.3% from precursor **43**, previously used by the authors in the synthesis of (−)-adalinine [49]. The spectral data (^1^H NMR, ^13^C NMR, MS) were identical to those of the natural product, and the specific rotation [α]_D_^28^ −11.4 (c 0.7, CHCl_3_) comparable to that found in the literature {[α]_D_^20^ −13 (CHCl_3_), [17]}.

#### Yu synthesis – 2009

Yu et al. prepared (−)-adaline (**1**) and the nonnatural enantiomer (−)-euphococcinine (**2**) in a 6-step sequence from 3,4-dihydro-2-ethoxy-2*H*-pyran (**58**) [50].

**Scheme 7:**
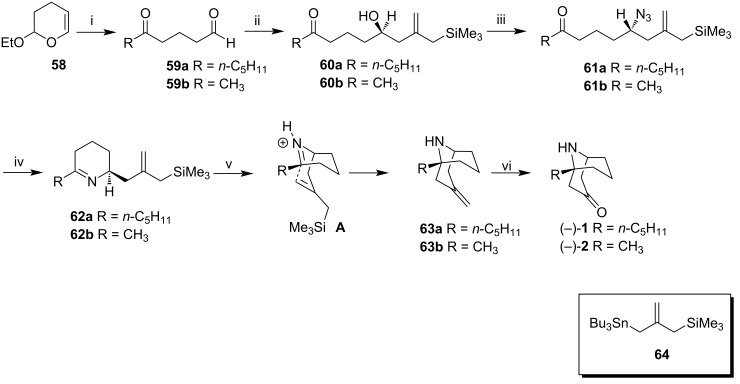
Synthesis of (−)-adaline (**1**) and (−)-euphococcinine (**2**). Reagents and conditions: i) 1. BuLi, *t*-BuOK, TMEDA, pentane, −78 °C to −20 °C; 2. RI, THF; 3. H_3_O^+^; 44% (R = *n*-C_5_H_11_) and 61% (R = CH_3_) over three steps; ii) **64**, (*S*)-BINOL-Ti^IV^[OCH(CF_3_)_2_]_2_ (5 mol %), PhCF_3_, −20 °C, 84% (97% ee, R = *n*-C_5_H_11_) and 94% (90% ee, R = CH_3_); iii) DIAD, Ph_3_P, NH_3_, THF, 81% (R = *n*-C_5_H_11_) and 74% (R = CH_3_); iv) Ph_3_P, Et_2_O, 20 °C, 77% (R = *n*-C_5_H_11_) and 68% (R = CH_3_); v) 1. CF_3_SO_3_H (1.1 equiv), toluene, 0 °C; 2. Bu_3_SnF (1.2 equiv), toluene, 81% (R = *n*-C_5_H_11_) and 74% (R = CH_3_); vi) OsO_4_ (3 mol %), KIO_4_, THF/H_2_O 3:1, 64% (R = *n*-C_5_H_11_) and 71% (R = CH_3_).

Treatment of **58** with a mixture of butyllithium and potassium *tert*-butoxide in the presence of TMEDA and pentane, followed by reaction with the corresponding alkyl iodides in THF, and finally, acidic cleavage of the generated acetal provided aldehydes **59a** and **59b** (Scheme 7). The key step in this synthesis was the allylic transfer, conducted by the dropwise addition of **64** in PhCF_3_ at −20 °C to a mixture of **59a** and **59b** and the chiral catalyst *S*-BINOL-TiIV [OCH(CF_3_)_2_]_2_ providing alcohols **60a** and **60b**, after 12 h at −20 ºC. In addition to the good yields in this step, both intermediates were obtained with excellent enantiomeric excesses (97% for R = *n*-C_5_H_11_ and 90% for R = CH_3_).

Compounds **60a** and **60b** were converted to azido ketones **61a** and **61b** by Mitsunobu reaction, and then these azido ketones underwent cyclization to furnish tetrahydropyridines **62a** and **62b** after treatment with Ph_3_P at 20 °C in diethyl ether. **62a** and **62b** were converted to **63a** and **63b** through an intramolecular allylic transfer reaction. After several attempts to perform this cyclization, the best conditions found were by using 1.1 equivalents of trifluoromethanesulfonic acid in toluene. After 5 minutes, 1.2 equiv of tributyltin fluoride was added to intermediate **A**, and at the end of the process, **63a** and **63b** were obtained after chromatographic purification with 81% yield for **63a** and 74% yield for **63b**. Finally, the alkenes were oxidized in the presence of osmium tetroxide and potassium periodate, to provide (−)-adaline (**1**) and (−)-euphococcinine (**2**).

Originally, Yu et al. synthesized (−)-adaline (**1**) with good yields and high enantiomeric excess using catalytic asymmetric allylation from commercially available **58**. Additionally, intramolecular allylic transfer led to the enatiopure azabicycles. This 6-step sequence was successfully completed and (−)-adaline (**1**) and (−)-euphococcinine (**2**) were prepared in overall yields of 11.9% and 15.2%, respectively. Specific rotation measured for (−)-adaline (**1**) was [α]_D_^20^ −12.2 (*c* 1.1, CHCl_3_); {lit. [α]_D_^20^ −11 (c 2, CHCl_3_), [17]} and for (−)-euphococcinine (**2**) was [α]^20^_D_ −6.1 (*c* 1.3, MeOH); {lit. [α]_D_ +6 (c 2.0, MeOH), for natural (+)-euphococcinine (**2**) [19]}.

#### Liebeskind synthesis – 2009

Liebeskind et al. prepared (−)-adaline (**1**) from the 5-oxopyridinylmolybdenum complex **66** [51]. This complex was developed as an organometallic enantiomeric scaffold for an asymmetric construction of a wide variety of heterocyclic systems.

**Scheme 8:**
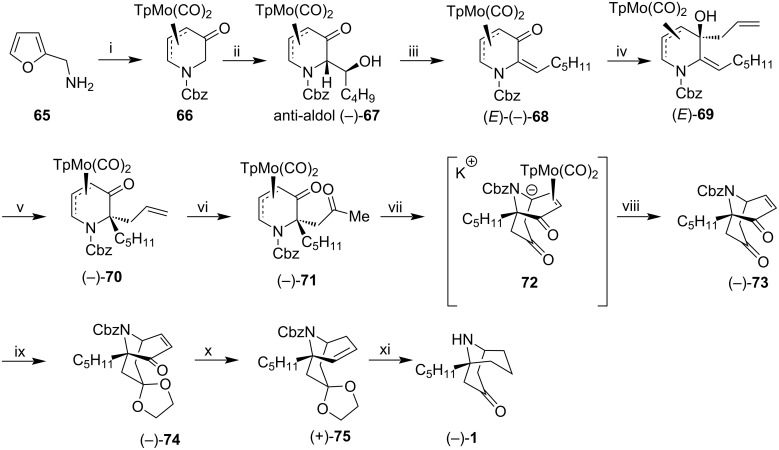
Synthesis of (−)-adaline (**1**). Reagents and conditions: i) Ref. [52]; ii) Et_3_N, TBDMSOTf, CH_2_Cl_2_, 0 °C to −78 °C, then valeraldehyde, TiCl_4_, CH_2_Cl_2_, 70%, 97.3% ee; iii) 1. DMAP, Et_3_N, MsCl, CH_2_Cl_2_; 2. DBU, CH_2_Cl_2_, 70% over two steps; iv) CH_2_=CHCH_2_MgCl, THF; v) HCl (4.0 M in dioxane), CH_2_Cl_2_, 78% over two steps; vi) PdCl_2_, CuCl, DMF/H_2_O (20:1), 23 °C, 93%; vii) KOSiMe_3_, 23 °C, 35 min; viii) NOPF_6_, DME, 80% over two steps; ix) 2-butanone ethylene acetal, cat. (CH_2_OH)_2_, BF_3_·Et_2_O, 85%; x) 1. Luche reduction, 98%; 2. a) NaH; b) CS_2_, c) MeI, 88%; 3. Bu_3_SnH, AIBN, heat, 75%; xi) 1. cat. Pd(MeCN)_2_Cl_2_, wet acetone, 95%; 2. H_2_, Pd-C, 90%.

The synthetic precursor **66** was obtained from furfurylamine (**65**), as previously published by the authors [52]. **66** was converted to the (*E*)-(−)-6-alkylidene-5-oxo **68** through a sequence of Mukayama aldol–dehydration reactions, via intermediate *anti*-aldol (−)-**67** (Scheme 8). The addition of Grignard reagent to the enone (*E*)-(−)-**68** occurred *anti* to the group TpMo(CO)_2_ to give adduct (*E*)-**69**, which was used in the next step without purification. The treatment of this adduct with HCl in dioxane promoted stereospecific semipinacol rearrangement in 78% yield over two steps. The resulting terminal alkene (−)-**70** was submitted to Vacker's conditions to produce methyl ketone (−)-**71** in 93% yield. The treatment of this ketone with potassium trimethylsilanolate induced a 1,5-Michael type reaction, via attack of tethered potassium enolate to neutral η3-allylmolibdenum.

The crude anionic intermediate **72** was treated with nitrosonium hexafluorophosphate in DME to provide bicyclic enone (−)-**73** with 80% yield over two steps. Protection of the non-conjugated ketone (−)-**73** as an acetal derivative occurred selectively to provide enone (−)-**74,** which was subjected to Luche reduction followed by removing the resulting alcohol under Barton–McCombie conditions, providing alkene (−)-**75** in 55% yield from (−)-**73**. Finally, the acetal group of (+)-**75** was hydrolyzed in the presence of catalytic Pd(MeCN)_2_Cl_2._ The intermediate ketone was subjected to simultaneous catalytic hydrogenation and hydrogenolysis of the protecting group Cbz to give (−)-adaline (**1**) in 90% yield.

The asymmetric synthesis achieved by Liebeskind et al. presented a high enantiomeric excess and good yields. Also, the proposed route differed from the previously mentioned in terms of common intermediaries. Therefore, it’s a new synthesis of (−)-adaline (**1**) and might eventually be applied to related homotropanes. In conclusion, (−)-adaline (**1**) was obtained in 14 steps from **66** using a new scaffold-based semipinacol/1,5-Michael-like strategy. The enantiomeric excess for precursor (−)-**75** was 97.6%, determined by HPLC. The overall yield for this synthesis was 13.4% and specific rotation [α]_D_^25^ −13.0 (*c* 0.73, CHCl_3_) {lit. [α]_D_ −13 (CHCl_3_), [17]}.

#### Spino synthesis – 2009

Spino et al. synthesized both (−)-adaline (**1**) and (+)-euphococcinine (**2**) [53]. The main features in this approach consisted of a 3,3-sigmatropic rearrangement to give an all-carbon quaternary center, a ring-closing alkene metathesis to give an 8-membered ring, and the use of a single enantiomer of *p*-menthane-3-carboxaldehyde to make two natural alkaloids of opposite configuration.

**Scheme 9:**
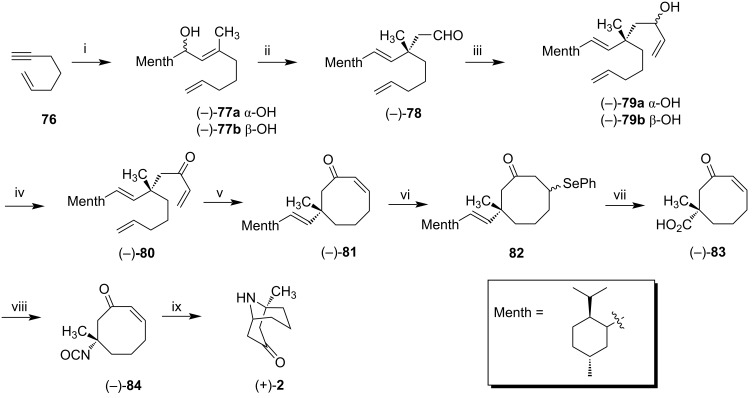
Synthesis of (+)-euphococcinine (**2**). Reagents and conditions: i) 1. Cp_2_ZrCl_2_,AlMe_3_, CH_2_Cl_2_; 2. *p*-menthyl-3-carboxaldehyde, 9:1 **77a**/**77b**, 67% (after chromatographic separation); ii) butyl vinyl ether, Hg(OAc)_2_, (10 mol %), sealed tube, 130–135 °C, 79%; iii) vinylmagnesium bromide, −78 °C; iv) Dess–Martin periodinane, 83% over two steps; v) Grubbs catalyst 2nd gen., CH_2_Cl_2_, reflux, 74%; vi) 1. (PhSe)_2_, NaBH_4_, EtOH; 2. EtOH/THF, 99%; vii) 1. O_3_, CH_2_Cl_2_, −78 °C; 2. DMS, −78 °C to rt.; 3. NaClO_2_, Na_2_HPO_4_, *t*-BuOH/H_2_O, 2-methy-2-butene, 90%; viii) DPPA, Et_3_N, toluene, reflux, 65%; ix) 1. CuCl, H_2_O/THF, rt to 40 °C; 2. aq K_2_CO_3_, rt, 63%.

Firstly, (+)-euphococcinine (**2**) was synthesized from terminal alkyne **76** (Scheme 9). This alkyne was prepared from 5-bromopentene, according to the procedure described by Negishi [54]. Zr-catalyzed carboalumination furnished vinylalane, treated with *p*-menthane-3-carboxaldehyde providing the allylic alcohols (−)-**77a** and (−)-**77b** in a 9:1 ratio. After chromatographic separation, alcohol (−)-**77a**, isolated in 67% yield and >99% de was subjected to a Claisen rearrangement, leading to aldehyde (−)-**78** in 79% yield (96% de determined by ^1^H NMR). (−)-**78** was treated with vinylmagnesium bromide to give a mixture of allyl alcohols (−)-**79a** and (−)-**79b,** which were oxidized to enone (−)-**80**. The enone (−)-**80** was subjected to ring-closing metathesis with Grubbs second-generation catalyst resulting in cyclic enone (−)-**81** in 74% yield. (−)-**81** was treated with phenylselenol to generate selenide **82** in high yield. The ozonolysis of **82** was accomplished, followed by the reductive workup of the resulting selenoxide and an increase in its temperature, eliminating selenoxide to generate carboxylic acid (−)-**83** in 90% yield. This acid was subjected to Curtius rearrangement [55] in the presence of DPPA as a source of azide, providing isocyanate (−)-**84** in 65% yield and complete stereochemistry retention. When isocyanate (−)-**84** was treated with copper chloride in water and THF, the (+)-euphococcinine (**2**) was obtained in 63% yield.

**Scheme 10:**
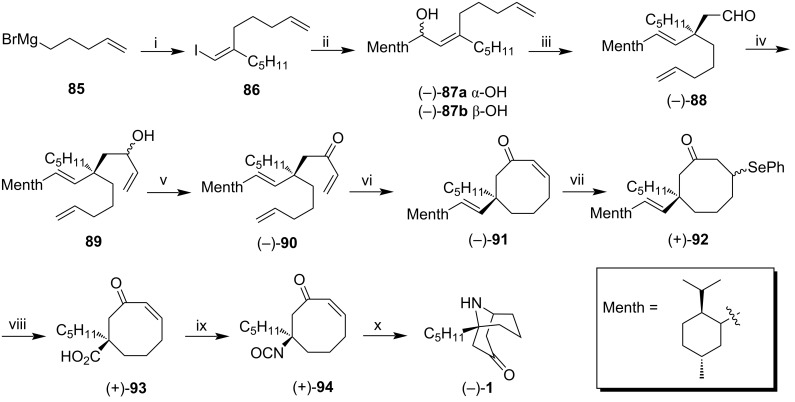
Synthesis of (−)-adaline **1**. Reagents and conditions: i) 1. CuBr^.^DMS, Et_2_O/DMS, -42 ºC; 2. 1-heptyne; 3. I_2_, THF, 82%; ii) 1. *n*-BuLi, Et_2_O, −78 °C to 0 °C; 2. *p*-menthyl-3-carboxaldehyde, 5:1 (−)-**87a** / (−)-**87b**, 50% (after chromatographic separation); iii) butyl vinyl ether, Hg(OAc)_2_, (10 mol %), sealed tube, 130–135 °C, 79%; iv) vinylmagnesium bromide, −78 °C; v) Dess–Martin periodinane, 83% over two steps; vi) Grubbs catalyst 2^nd^ gen., CH_2_Cl_2_, reflux, 74%; vii) 1. (PhSe)_2_, NaBH_4_, EtOH; 2. EtOH/THF, 99%; viii) 1. O_3_, CH_2_Cl_2_, −78 °C; 2. DMS, −78 °C to rt; 3. NaClO_2_, Na_2_HPO_4_, *t*-BuOH/H_2_O, 2-methy-2-butene, 90%; ix) DPPA, Et_3_N, toluene, reflux, 65%; x) 1. CuCl, H_2_O/THF, rt to 40 °C; 2. aq K_2_CO_3_, rt, 63%.

A similar sequence was used to synthesize natural (−)-adaline (**1**, Scheme 10). In this case, vinyl iodide **86** was obtained from the carbocupration of **85**, a Grignard derivative of 1-heptyne [56]. After a lithium–halogen exchange, the corresponding vinyllithium was treated with *p*-menthane-3-carboxaldehyde to give the isomeric allylalcohols (−)-**87a** and (−)-**87b** in a 5:1 ratio. After chromatographic separation, (−)-adaline (**1**) was obtained from pure (−)-**87a**, using the same sequence described previously for (+)-euphococcinine (**2**) with similar yields.

Through this methodology, (+)-euphococcinine (**2**) was obtained in 13 steps from **76**, in an overall yield of 11.7% and specific rotation [α]_D_^20^ +5.4 (*c* 0.65, MeOH); {lit. [α]_D_ +7.5 (*c* 2.0, MeOH), [26]}. Also, (−)-adaline (**1**) was acessed from **85** in 16 steps, and specific rotation [α]_D_^20^ = −11.2 (*c* = 0.60, CHCl_3_); {lit. [α]_D_ –11 (*c* 2.0, CHCl_3_), [26]}. It is worth mentioning that Spino et al. elegantly proposed a menthol derivative as a chiral auxiliary for the synthesis of (−)-adaline (**1**) and (+)-euphococcinine (**2**). The cyclooctatetraene derived selenides **82** and (+)-**92** are, at some point, similar to the one obtained by Renbaun in the (−)-adaline (**1**) synthesis. Renbaun generated the quaternary center by adding a chiral amine to the cyclooctatetraene system. On the other hand, Spino et al. firstly made the quaternary center, followed by cyclization. Furthermore, Spino's synthesis involved key reactions such as Claisen rearrangement, olefin metathesis, and the Curtius rearrangement that allowed both natural products in good yields.

#### Davis synthesis – 2010

Davis and Edupuganti prepared the (−)-adaline (**1**) and the non-natural isomer (−)-euphococcinine (**2**) through a four-step intramolecular Mannich cyclization cascade reaction [57]. In this methodology, the alkaloids were prepared by treating the convenient *N*-sulfinylamino ketone ketal precursor on heating with NH_4_OAc:HOAc.

**Scheme 11:**
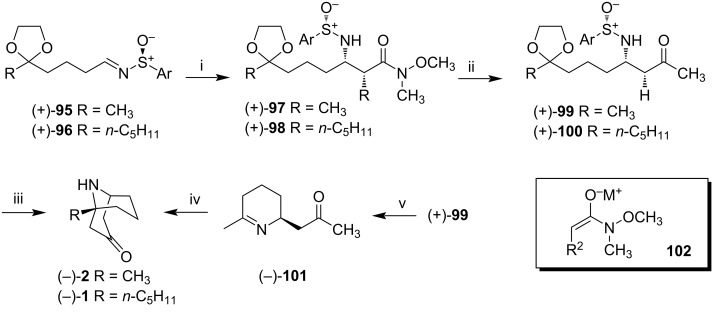
Synthesis of (−)-euphococcinine (**2**) and (−)-adaline (**1**). Reagents and conditions: i) **102**, KHMDS, Et_2_O, −78 °C, 73% (96:4 de; R = CH_3_) and 71% (95:5 de; R = *n*-C_5_H_11_); ii) CH_3_MgBr, −78 °C, THF; 94% (25:1 de; R = CH_3_) and 95% (22:1 de; R = *n*-C_5_H_11_); iii) NH_4_OAc/HOAc/EtOH, 75 °C, 36 h, (90%, R = CH_3_) and 3.5 days (85%; R = *n*-C_5_H_11_); iv) NH_4_OAc/HOAc/EtOH, 75 °C, 36 h, 93%; v) 3 N HCl, MeOH, THF, 86%.

Oxo-sulfinimes (+)-**95** and (+)-**96** were added to a −78 °C solution of the *N*-methoxy-*N*-methylacetamide enolate **102**, leading to Weinreb amides (+)-**97** and (+)-**98**, respectively, with good yields and high diastereoisomeric excesses (Scheme 11). The reaction of (+)-**97** and (+)-**98** with five equivalents of methylmagnesium bromide provided ketones (+)-**99** and (+)-**100**, in diastereoisomeric excess of about 92%. *N*-sulfinyl-β-aminoketone ketal (+)-**99** was subjected to Mannich cyclization, via treatment with 25 equivalents of ammonium acetate in acetic acid at 75 °C, generating (−)-euphococcinine (**2**) in 90% yield. A similar treatment to ketal (+)-**100** provided (−)-adaline (**1**) in 85% yield.

Ketal (+)-**99** was also subjected to the treatment with 3 N aqueous HCl in MeOH and THF, to provide the homotropane system directly; however, this reaction led to the piperideine ketone (−)-**101** in 86% yield. (−)-**101** was also submitted to the same conditions described previously (25 equiv of ammonium acetate in 1:1 HOAc/EtOH) to furnish (−)-euphococcinine (**2**) in 93% yield.

In this work, (−)-euphococcinine (**2**) and (−)-adaline (**1**) were obtained in three steps from oxo-sulfinimes (+)-**95** and (+)-**96**, in overall yields of 61.8% and 57.3%, respectively. The conversion of precursors *N*-sulfinylamino ketone ketals directly to the desired homotropanes represents a four-step intramolecular Mannich cyclization cascade reaction, being the most efficient method to date for the (−)-adaline (**1**) and (−)-euphococcinine (**2**) syntheses. Specific rotation was [α]_D_^20^ −6.4 (*c* 0.83, MeOH); {lit. [α]_D_^20^ −6.5 (*c* 1.80, MeOH), [26]} for (−)-euphococcinine (**2**) and [α]_D_^20^ −12.6 (*c* 0.85, CHCl_3_); {lit. [α]_D_ −13 (CHCl_3_), [17]} for (−)-adaline (**1**).

#### Kurti synthesis – 2020

Kürti et al. developed racemic *N*-methyleuphococcinine ((±)-**3**), exploring the use of arylboronic acids as catalysts for C-allylation of unprotected oximes with allyl boronates [58].

**Scheme 12:**
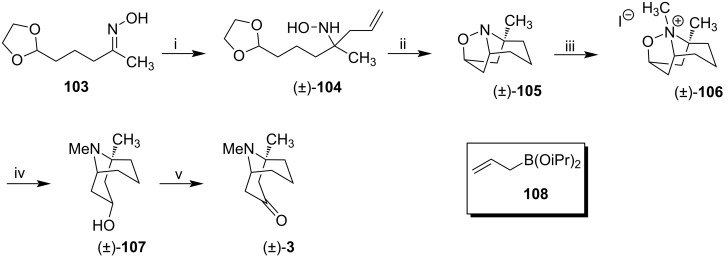
Synthesis of *N*-methyleuphococcinine **3**. Reagents and conditions: i) **108** (1.5 equiv), 3,5-di-F-C_6_H_3_B(OH)_2_ (0.1 equiv), DCE, 50 °C, 18 h, 95%; ii) 1. 1 M HCl, rt, 30 min; 2. PhMe, 100 °C, 10 h, 57% over two steps; iii) MeI, Et_2_O, 24 h, 73%; iv) Zn, AcOH/THF/H_2_O, 30 °C, 6 h, 75%; v) Dess–Martin periodinane, NaHCO_3_, THF, 0 °C to rt, 58%.

After screening to find the best reaction conditions, oxime **103** was converted to α-tertiary acetal-protected hydroxylamine (±)-**104** in the presence of 3,5-difluorophenylboronic acid and diisopropyl allyl boronate (**108**) in 95% yield after 18 h (Scheme 12). Hydroxylamine (±)-**104** was hydrolyzed in the presence of aqueous hydrochloric acid, and the resulting nitrone was heated in toluene to yield the homotropane (±)-**105** in 57% yield over two steps.

(±)-**105** was alkylated with excess methyl iodide to form the corresponding ammonium salt (±)-**106** in 73% yield. The N–O bridge of ammonium salt (±)-**106** was reduced with zinc. The resulting diastereomerically pure amino alcohol (±)-**107** was then oxidized in the presence of Dess–Martin periodinane to deliver *N*-methyleuphococcinine ((±)-**3**).

Although Kurtis' synthesis was racemic, it presented a few steps and led to *N*-methyleuphococcinine ((±)-**3**) in good yields. Besides, arylboronic acids proved to be efficient catalysts for the C-allylation of unprotected oximes. Using this method, the authors accessed the racemic form of *N*-methyleuphococcinine (**3**) in 6 steps with a total yield of 17.2% from oxime **103**.

## Conclusion

The peculiar structural factors of homotropane alkaloids added to the intriguing biological activity exerted by ladybirds (as demonstrated in this review for *A. bipunctata*), besides the fact that the insect releases these substances in minimal quantities, make these targets highly relevant when considering total synthesis. Since Tursch's pioneering work, several total and formal syntheses of homotropane alkaloids released by coccinellids have been carried out, contributing to a more accurate chemical and biological understanding of these alkaloids. Specifically, in this review, the main points in the synthesis of coccinellid alkaloids are: i) dipolar cycloaddition; ii) olefin metathesis; iii) intramolecular Mannich reaction. Cyclization steps, summarized in Table 1, have shown to be efficient in the construction of an azabicyclononane system and also to provide enantiomerically pure alkaloids.

Therefore, homotropane-based compounds continue to attract the attention of researchers involved in the progress for new synthetic methodologies to reproduce these natural products and synthesize their analogs, improving existing methods.

**Table 1:** Summary of syntheses described in this review.

Synthesis	Products	Key step	Overall yield	Number of steps

Holmes [40], 1995	(±)-**1** and (±)-**2**	intramolecular dipolar cycloaddition	15.0–25.3% from **5** (or **6**)	8
Murahashi [41], 2000	(+)-**2** (−)-**1** precursor	intramolecular dipolar cycloaddition	2.1% from **17** 7.3% from **17**	7 5
Meyers [42], 2000	(+)-**2**	intramolecular Mannich reaction	51.2% from **26**	5
Ikeda [45], 2002	(±)-**2** precursor	Bu_3_SnH-mediated (radical) cyclization	14.3% from **34**	7
Kibayashi [48], 2002	(−)-**1**	ring-closing methathesis	28.3% from **43**	13
Yu [50], 2009	(−)-**1** (−)-**2**	intramolecular allylic transfer	11.9% from **58** 15.2% from **58**	8 8
Liebeskind [51], 2009	(−)-**1**	base-promoted cyclization	13.4% from **66**	14
Spino [53], 2009	(+)-**2** (−)-**1**	base-promoted cyclization	11.7% from **76** 7.3% from **85**	13 16
Davis [57], 2010	(−)-**2** (−)-**1**	intramolecular Mannich reaction	61.8% from **95** 57.3% from **96**	3 3
Kürti [58], 2020	*N*-Me-**3**	termal-promoted cyclization	17.2% from **103**	6
